# Performances and microbial features of an aerobic packed-bed biofilm reactor developed to post-treat an olive mill effluent from an anaerobic GAC reactor

**DOI:** 10.1186/1475-2859-5-16

**Published:** 2006-04-05

**Authors:** Lorenzo Bertin, Maria Chiara Colao, Maurizio Ruzzi, Leonardo Marchetti, Fabio Fava

**Affiliations:** 1 DICASM, Faculty of Engineering, University of Bologna, viale Risorgimento 2, I-40136 Bologna, Italy; 2 DABAC, University of Tuscia, Via C. de Lellis, snc. I-01100 Viterbo, Italy

## Abstract

**Background:**

Olive mill wastewater (OMW) is the aqueous effluent of olive oil producing processes. Given its high COD and content of phenols, it has to be decontaminated before being discharged. Anaerobic digestion is one of the most promising treatment process for such an effluent, as it combines high decontamination efficiency with methane production. The large scale anaerobic digestion of OMWs is normally conducted in dispersed-growth reactors, where however are generally achieved unsatisfactory COD removal and methane production yields. The possibility of intensifying the performance of the process using a packed bed biofilm reactor, as anaerobic treatment alternative, was demonstrated. Even in this case, however, a post-treatment step is required to further reduce the COD. In this work, a biological post-treatment, consisting of an aerobic biological "Manville" silica bead-packed bed aerobic reactor, was developed, tested for its ability to complete COD removal from the anaerobic digestion effluents, and characterized biologically through molecular tools.

**Results:**

The aerobic post-treatment was assessed through a 2 month-continuous feeding with the digested effluent at 50.42 and 2.04 gl^-1^day^-1 ^of COD and phenol loading rates, respectively. It was found to be a stable process, able to remove 24 and 39% of such organic loads, respectively, and to account for 1/4 of the overall decontamination efficiency displayed by the anaerobic-aerobic integrated system when fed with an amended OMW at 31.74 and 1.70 gl^-1^day^-1 ^of COD and phenol loading rates, respectively. Analysis of 16S rRNA gene sequences of biomass samples from the aerobic reactor biofilm revealed that it was colonized by *Rhodobacterales*, *Bacteroidales*, *Pseudomonadales*, *Enterobacteriales*, *Rhodocyclales *and genera incertae sedis TM7. Some taxons occurring in the influent were not detected in the biofilm, whereas others, such as *Paracoccus*, *Pseudomonas, Acinetobacter *and *Enterobacter*, enriched significantly in the biofilter throughout the treatment.

**Conclusion:**

The silica-bead packed bed biofilm reactor developed and characterized in this study was able to significantly decontaminate anaerobically digested OMWs. Therefore, the application of an integrated anaerobic-aerobic process resulted in an improved system for valorization and decontamination of OMWs.

## Background

Olive mill wastewater (OMW) is the effluent resulting from olive oil producing processes. Due to their high COD loading rates and content of toxic phenolic compounds, OMWs have to be decontaminated before being discharged [1,2]. Among the treatment methods currently available for this effluent, anaerobic digestion is generally considered the most promising because of its ability to combine a marked OMW decontamination potential with the ability to generate biogas rich of CH_4 _[3,4]. The large scale anaerobic digestion of OMWs is normally conducted in dispersed-growth reactors, where however the removal of the toxic phenolic fraction is often unsatisfactory [5,6]. The possibility of intensifying the dephenolisation potential of the process by performing it in anaerobic column reactor packed with granular activated carbon (GAC) was recently demonstrated [7,8]. GAC-digestor resulted to be a reproducible and stable OMW digesting process capable of a tolerance to high OMW organic loads and methanogenic performances significantly higher than those of the other bench-scale up-flow packed-bed biofilm OMW digestors described so far in the literature [3,9-11] and of those of the dispersed-growth digestors previously developed with the same microbial inoculum [7,12,13]. Molecular analysis of GAC-packed bed biofilm showed that it was heterogeneously composed by a large number of *Proteobacteria*, bacteria of the *Flexibacter-Cytophaga-Bacteroides *group, sulfate-reducing bacteria and low G+C gram-positive bacteria along with a limited number of highly abundant *Archaea *taxons mostly due to *Methanobacterium formicicum *[8].

Despite of its improved OMW decontamination performances, GAC-digestor generated effluents with a COD generally too high to allow their release in the environment or their use in agriculture [14]. Thus, the opportunity to implement the GAC-digestor decontamination potential by integrating it with an aerobic biological post-treatment was explored in this study. A "Manville" silica bead (SB)-packed bed aerobic reactor was developed, hydraulically connected to the GAC-digestor and employed to post-treat OMW digested effluents. The integrated anaerobic-aerobic process was operated in continuous mode and assessed through a 2 month experiment. In this paper the decontamination potential and main microbial features (e.g., structure and spatial distribution of reactor microbial community) of the aerobic post-treatment are reported and discussed. Despite the large number of studies already published on the biotreatment of OMWs [2-4,7,8,11-13,15], this is the first work in which the use of an integrated anaerobic-aerobic continuously operating biofilter system is proposed for such a purpose. Furthermore, this is also the first work in which an aerobic biofilter colonized by the native microflora of an anaerobically digested OMW is assessed under a technological and biological point of view.

## Results

### Performances of the integrated anaerobic-aerobic biofilter system

The integrated anaerobic-aerobic system was fed with AOMW at high and constant organic loading rate for a 2 month period (Table 1). Fig. 2 shows the evolution of COD and total phenol concentration in the influent of the integrated system and in the influent and the effluent of the aerobic biofilter throughout the whole experiment. The pollutant removal attained with the anaerobic digestion (expressed as COD or phenol removal efficiency, and calculated by dividing the amount of pollutant removed by the amount of pollutant occurring in the reactor influent) was of about 45 and 60% of initial AOMW COD and phenolic compounds, respectively. These removal efficiency were improved by about 30% through the aerobic post-treatment, thus permitting an overall removal of AOMW COD and phenol loading rates applied to the sequential anaerobic-aerobic treatment of about 59 and 76%, respectively (Table 1). No HPLC-detectable aromatic metabolites accumulated in the aerobic SB-biofilter throughout the whole experiment. A large array of volatile fatty acids (acetate, propionic acid, iso-butyric acid, butyric acid and valeric acid) occurred in the SB-reactor influent, where they were responsible for about 30% of the influent COD, but they were not detected in the reactor effluent. pH and redox potential of the SB-reactor influent were 5.2 ± 0.2 and -290 mV, respectively, but they increased markedly (up to 6.5 and 150 mV, respectively) as a result of the aerobic treatment.

**Figure 1:**
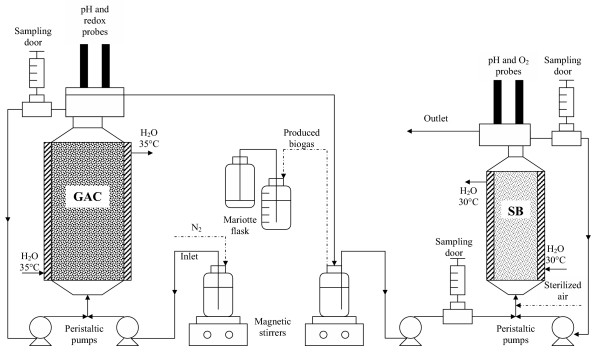
**Integrated anaerobic-aerobic biofilter system**. Scheme of the anaerobic-aerobic packed bed reactor system developed in the study.

**Figure 2:**
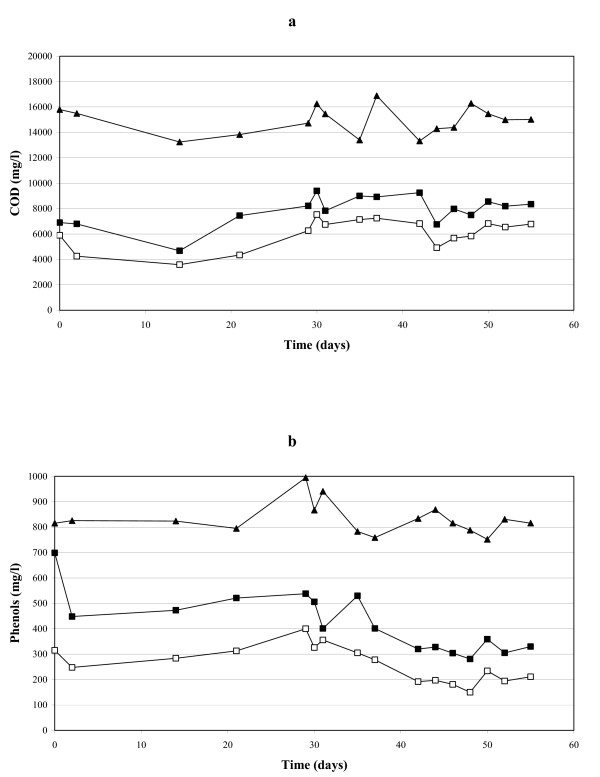
**COD and phenol removals**. Profiles of COD (a) and total phenol concentration (b) in the influent (▲) of the integrated anaerobic-aerobic process, in the influent (■) and the effluent (□) of the aerobic SB biofilter continuously fed with AOMW throughout the 56 days of treatment. Data provided were obtained through double measurements.

**Table 1:** COD and phenol removal efficiency. COD and total phenol loading rates with which the reactors were fed along with removal efficiency (%) of COD and total phenol biodegradation occurred both in the sole reactors and in the whole integrated anaerobic-aerobic system in steady state conditions.

	Experiment duration	Influent COD	Influent Total phenol concentration	D	COD Loading rate	Total phenol Loading rate	Effluent COD	Effluent Total phenol concentration	COD Removal efficiency (%)	Total phenol Removal efficiency (%)
	(day)	(gl^-1^)	(gl^-1^)	(day^-1^)	(gl^-1^day^-1^)	(gl^-1^day^-1^)	(gl^-1^)	(gl^-1^)		
GAC-digestor	55	15.28 ± 1.45	0.82 ± 0.13	2.077	31.74 ± 3.01	1.70 ± 0.28	8.35 ± 0.75	0.33 ± 0.04	45	60
SB-aerobic reactor	55	8.17 ± 0.77	0.33 ± 0.04	6.171	50.42 ± 4.78	2.04 ± 0.27	6.24 ± 0.69	0.20 ± 0.03	24	39

Integrated System	55	15.28 ± 1.45	0.82 ± 0.13				6.24 ± 0.69	0.20 ± 0.03	59	76

### Biological features of the SB-biofilter

The biological properties of the SB aerobic biofilter were investigated at the end of the 2 months of operation by determining its content of immobilized biomass and the structure and spatial distribution of its microbial community. The amount of immobilized biomass detected at 5, 20 and 38 cm of height (from the bottom) of the reactor packed-bed was (in mg of dried biomass/g of dried support) 12.74 ± 0.85, 11.38 ± 1.01 and 27.51 ± 1.58, respectively. Running the average of such values (17.21 mg/g) and considering that the reactor was packed with 0.213 kg of dried support, it can be estimated that the SB-reactor harbored a total immobilized biomass of 3.67 g (on dry weight basis).

**Figure 3:**
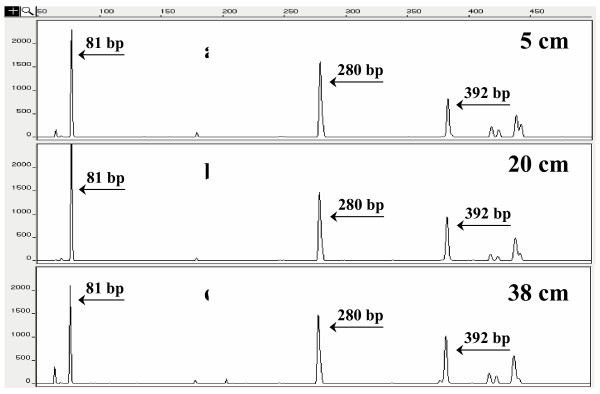
**Biofilm electropherograms**. Electropherogram of the 5' T-RFs derived from *Rsa*I digestion of the *Bacterial *community 16S rDNA of the biofilm samples taken at different region of the reactor (a – 5 cm, b – 20 cm, c – 38 cm height).

The structure of the microbial community occurring at different regions of the reactor packed-bed as well as in the influent and effluent of the reactor, was investigated through T-RFLP analysis. Fluorescent amplifications of 16S rRNA genes with universal eubacterial primers were successful for all of the samples examined. T-RFLP analysis of biofilm consortia obtained at 5, 20 and 38 cm height of the reactor packed bed yielded the same three major T-RFs of 81, 280 and 392 bp length with *Rsa*I restriction enzyme digestion. The three biofilm samples displayed similar T-RFLP patterns with a predominant T-RF with 81 bp of length (Fig. 3). Also the abundance of each bacterial population (represented as the peak height of each T-RF in relation to the total peak height of all T-RFs detected) was almost identical in the three biofilm samples. The major T-RFs detected in the influent after *Rsa*I digestion were 98, 280 and 386 bp in length (Table 2). These peaks, which were detected and characterized in the effluent of the GAC reactor [8], were affiliated with *Synergistes *(clone B12, T-RF of 98 bp), *Bacteroides *(clone B25, T-RF of 280 bp) and γ-*Proteobacteria *(clone B1, T-RF of 386 bp), respectively. Only T-RF of 386 bp in length was detected in the AOMW introduced in the integrated biofilters. A microbial community displaying T-RFLP patterns very similar to those obtained from biofilm was found to occur in the SB-reactor effluents (data not shown).

**Table 2:** Results of T-RF analyses. Schematic representation of T-RFs obtained after RsaI digestion of 16S rRNA genes amplified from DNA of samples of OMW, anaerobically-treated OMW (GAC-reactor effluents) and biofilm from the SB-aerobic biofilter.

	**T-RF length (bp)**														
**Sample**	68	81	98	168	183	274	280	282	386	392	404	414	428	436	441
OMW									**94**			4	2		
SB-reactor influent (GAC-digestor effluent)			**80**	2		1	7	3	4		1			1	1
SB-reactor biofilm	2.6	**37.7**			1.7		26.4			13.5			3.6	7.3	4.5

The dominant (height) T-RF peak in each profile is indicated in boldface. Individual clones having a corresponding peak in the T-RF profiles are indicated below. The numbers indicate the relative abundance of individual T-RF. These values were calculated based on the peak height of individual T-RF in relation to the total peak height of all T-RFs detected in the respective community fingerprint pattern. The peak heights were automatically quantified by GeneScan software (PE Applied Biosystems), performing the analysis with a peak height threshold of 50 fluorescent units.

**Figure 4:**
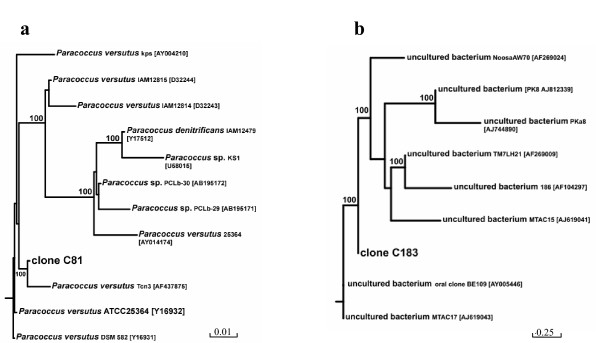
**Phylogenetic trees of 16S rRNA genes**. Phylogenetic trees of 16S rRNA genes belonging to *Paracoccus *(T-RF 81 bp; panel a) and genera incertae sedis TM7 (T-RF 183 bp; panel b) analyzed using bootstrap and the neighbor-joining methods as distance measures. The cloned sequences are indicated in boldface and the GeneBank accession numbers of sequences are in bracket. The distance bar is shown under the tree, and bootstrap values (1000 replicates) are given for selected nodes.

**Figure 5:**
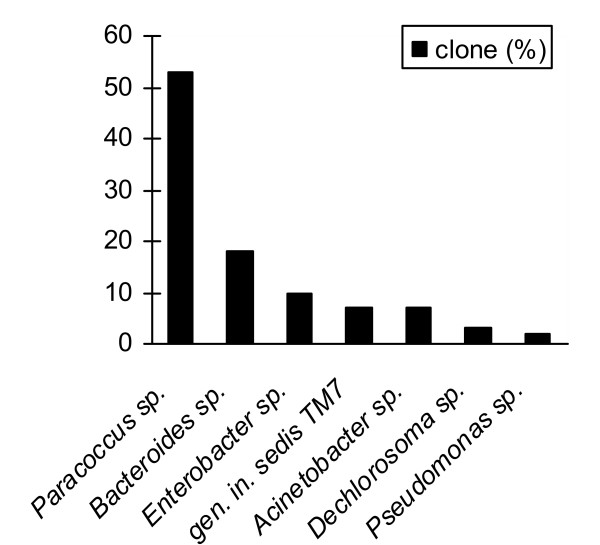
**Clone distribution**. Clone distribution based on the sequence of 16S rRNA genes recovered from clone libraries.

A total of 34 clones from 16S rRNA gene clonal libraries of biofilm consortia were randomly picked for PCR amplification and the amplified products digested with *Eco*RI, *Eco*RI plus *Kpn*I and *Rsa*I restriction enzymes. Eight different types of RFs (C81, C183, C280, C392, C610, H172, PS1 and PS156) were obtained (data not shown). Sequencing analysis of these RFs showed that clones from biofilm samples taken at different regions of the reactor having the same RF pattern were identical. The corresponding rDNA inserts were considered to belong to the same sequence type, resulting in the identification of 8 operational taxonomic units (OTUs). The sequences retrieved from the biofilm libraries were compared with the 16S rRNA reference sequences of the Ribosomal Database Project II database and were found to be > 80% identical to known rDNA sequences. Clone C280 was identical to clone B25 (*Bacteroides *group; Accession number AJ608923), which was detected in the effluent of the GAC reactor [8]. Clone C81 was closely related to *Paracoccus versutus *type strains ATCC 25364 (Accession number Y16962) and DSM 582 (Accession number Y16931) with 97.2% similarity (Fig. 4 panel A) and to *Paracoccus *spp. strains isolated from a solid-phase denitrification process using poly(ε-caprolactone) as carbon and energy source [16] with 97.6% similarity. The remaining six clonal sequences loosely related to *Pseudomonadales *(clone H172 with 80.7% similarity to *Acinetobacter schindleri *type strain LUH5832; clone PS1 with 80.7% similarity to *Pseudomonas nitroreducens *type strain LMG 1224; clone PS156 with 96.4% similarity to *Pseudomonas alcaliphila *type strain AL15–21), *Enterobacteriales *(clone C392 with 75% similarity to *Enterobacter cloacae*), *Rhodocyclales *(clone C610 with 94.0% similarity to *Dechlorosoma *spp. strain PCC) and genera incertae sedis TM7 (clone C183 with 89.5% similarity to uncultured bacterium TM7 LH21, Fig. 4 panel B). Analysis of clone distribution indicated that *Paracoccus *and *Bacteroides *were the major groups in the biofilm consortia obtained from the different portions of the reactor packed-bed (70%, Fig. 5).

T-RFLP analysis of each RF showed that clone C81 could be matched with the 81 bp length fragment after *Rsa*I digestion and clone C280 could be matched with the 280 bp length *Rsa*I-fragment. In addition, clone C81 generated a small T-RF with the size of 385 bp after *Rsa*I digestion due to incomplete digestion. Clone C183 generated a 183 bp length fragment which was a low abundant T-RF in the T-RFLP patterns of biofilm consortia. Clone C392 generated T-RF with the size of 392 bp, while clone H172 did not generate a *Rsa*I T-RF.

## Discussion

An intensified OMW anaerobic digesting process consisting of a GAC-packed bed biofilm reactor was recently developed and assessed [8]. In order to further reduce COD of the effluents resulting from the process, the possibility of implementing the GAC-digestor with a tailored aerobic post-treatment was explored in this study. Integrated anaerobic-aerobic treatments have been often proposed for a high rate, cost effective and sustainable disposal of agroindustrial effluents [17-22]. However, a little is known [13,23] about the feasibility of this approach in the management of OMWs. In the present study, a SB-packed bed biofilter was selected to post-treat digested OMW as the same technology was recently found to be very effective in the final decontamination of a similar anaerobically digested wastewater [23]. However, differently from this previous work, where it was inoculated with a defined co-culture of specialised bacteria and applied in discontinuous mode [23], SB-biofilm reactor was here hydraulically connected to the OMW digestor and allowed to be colonized by the influent native microflora able to grow on the SB surface under the aerobic conditions provided. To the very best of our knowledge, this is the first integrated anaerobic-aerobic biofilm system developed so far for an improved, continuous biomethanization and decontamination of OMWs.

The integrated biofilter system was assessed through a 2 month experiment performed under open mode of operation by feeding it with a high and constant AOMW organic loading rates. It was found to remove about 59 and 76% of COD and phenol loading rates, respectively. The aerobic post-treatment contributed for about 1/4 of such removal efficiency, and these performances were comparable to those displayed on a similar anaerobically digested effluents by the aerobic conventional activated sludge post-treatment developed by Beccari et al. [13] and lower than those observed by Bertin et al. [8] with a similar aerobic SB-biofilm reactor. However, it has to be pointed out that the latter two processes were operated at lower COD and phenol loading rates and under batch mode.

SB-reactor biofilm was found to consist of *Rhodobacterales*, *Bacteroidales*, *Pseudomonadales*, *Enterobacteriales*, *Rhodocyclales *and genera incertae sedis TM7. T-RFLP analysis and 16S rRNA gene cloning indicated that *Paracoccus *and uncultured strain B25 were the major groups occurring at different depths of the biofilter. Strain B25, an anaerobic bacterium of the *Bacteroides *group, was one of the major strains colonizing the GAC anaerobic digestor [8], whose effluents were used to feed the SB-aerobic biofilter. Instead, other members of the same group occurring in the anaerobic GAC biofilm digestor (i.e., uncultured bacteria B23 and B24) [8], were not found in the SB-biofilter. The presence of anaerobic *Bacteroides *in aerobic reactors has been already reported in the literature. The occurrence of these bacteria has been documented in conventional aerobic reactors treating municipal wastewaters [24] or anaerobically pre-treated tert-butyl alcohol-contaminated wastewaters [25], as well as in packed-bed biofilm reactors developed for the oxidization of sulfide-containing effluents [26]. Members of the *Bacteroides *group are often able to degrade various refractory biomacromolecules, such as cellulose, chitin, DNA, lipids, and proteins, which generally are abundant in a biofilm, in which dead microorganisms are trapped. These species are also known for their ability to produce exopolysaccharide slime, that generally has a primarily role in biofilm formation and development [27]. Thus, as suggested by Ferrera et al. [26], the presence of *Bacteroides *strains in aerobic biofilm reactors might be correlated to their role in the biofilm development and in the long-term functioning and versatility of the process. The abundant occurrence of the *Paracoccus *sp. T-RF clone C81 in the biofilm is also interesting because *Paracoccus *is a quite biochemical versatile genus, able to display a wide range of degradative capabilities. Some *Paracoccus *strains are capable of aerobic denitrification (simultaneous reduction of oxygen and nitrate) and heterotrophic nitrification (oxidation of ammonium to nitrite during heterotrophic growth), whereas other strains are capable of a) aerobic growth on formate, b) aerobic chemolithoautotrophic growth using carbon disulfide as energy sources, c) methylotrophic growth on methanol or d) heterotrophic growth on diethyl sulfide, thioethanol, thioacetic acid or substituted thiophenes. Some other strains of this group can also grow anaerobically using thiosulfate, carbon disulfide, methanol or formate as energy sources and nitrate as final electron acceptor [28]. The classification of the genus *Paracoccus *(alpha subgroup of the *Proteobacteria*) has undergone serious changes during the past decade [29]. Several new species have been isolated and, currently, the genus consists of 17 species, which can be found in different environments, including soil [30], contaminated groundwater [31], biofilters [32], sewage sludge [33], denitryfing reactors [34] and industrial wastewaters [35]. Bacteria belonging to the genus *Paracoccus *are important components of many wastewater treatment system communities [36]. Most species in the genus can use nitrate and its reduction products as an alternative electron acceptor to oxygen during anaerobic respiratory growth [37,38], and, therefore, can survive and proliferate in ecosystems with fluctuating aerobic/anaerobic conditions. In our study, 16S rDNA sequences belonging to *Paracoccus *populations (T-RF 81 bp) were not detectable in the AOMW that entered the integrated process and in the SB-reactor influent. (Table 2). These bacteria were therefore massively enriched in the aerobic biofilm throughout the treatment to become the dominant eubacterial group. This finding might be ascribed to the ability of some *Paracoccus *species to denitrify in the presence of oxygen up to levels of 90% of air saturation [39,40], ability that might allow the same strains to use both oxygen and nitrate as terminal electron acceptors [39] and therefore to have nutritional advantages that, in turn, might have allowed them to extensively colonize the reactor system developed in this study exposed to varying oxygen concentrations. The clonal analysis of the 16S rRNA genes suggests that the microbial consortia that inhabit the aerobic biofilter also included minor members belonging to the uncultivated bacterial division TM7 (Fig. 4, panel B). Candidate division TM7 has no cultivated representatives and has been exclusively characterized by environmental sequence data. This division takes its name from the German peat bog from which the first sequence was obtained [41], but additional TM7 sequences deposited by several other investigators have demonstrated that members of this division are present in extremely diverse environments, including soil, freshwater, seawater, hot springs, mouse feces, termite guts, activated sludges and in human subgingival plaque samples [42-44]. In situ analysis revealed that members of the uncultivated TM7 division are capable of surviving and growing under a wide range of conditions. Ouverney et al. [45] suggested that TM7 members may be involved in the formation of a scaffold or biofilm, which could support the development of a disease-associated microbial community in human plaque. Thus, the evidence that TM7-like 16S rDNA could be amplified from the samples taken at different regions of the reactor let to speculate that TM7-related bacteria have had a role in the SB-biofilm formation. Unfortunately, the current limited knowledge on this uncultivated bacterial division does not allow us to speculate on the contribution that TM7 bacteria might have had on the aerobic decontamination of the anaerobic digested AOMW.

Other bacteria occurring in the biofilm consortia consisted of phylotypes affiliated with *Pseudomonas, Acinetobacter *and *Enterobacter *genera, which are common members of aerobic microbial consortia involved in the biodegradation of biogenic and xenobiotic compounds [46-48]. These members of *Pseudomonadales *and *Enterobacteriales *were not detected in the effluents of the anaerobic digestor and therefore enriched in the reactor throughout the 2 months-experiment. This suggests that they were significantly involved in removing organic compounds occurring in the anaerobically digested influent of the SB-aerobic reactor.

## Conclusion

In conclusion, an effective aerobic biofilm technology able to significantly decontaminate anaerobically digested OMWs was developed, integrated with the anaerobic digestor and assessed in this study. To the very best of our knowledge, this is the first report in which a similar biofilm process is proposed for such a purpose and assessed through an integrated chemical and molecular biotechnology monitoring.

## Methods

### Chemicals and OMWs employed

Chemicals employed in the analysis of COD, total phenols as well as solvents used for HPLC and ion chromatography were obtained from Sigma-Aldrich (Milan, Italy) and Baker Italia (Milan, Italy). "Manville" silica spherical beads (diameter: 5 mm) (SB) were supplied by Manville Filtration and Minerals (Denver, CO, USA).

An industrial OMW containing about 30 g/l of COD and 2.0 g/l of total phenols was collected from an Italian olive oil producing plant, stored in filled and sealed plastic jars at 4°C and employed to prepare an amended OMW, AOMW, that was then used in the study. AOMW was prepared from the industrial OMW by a) diluting the latter with an equal volume of tap water, b) amending the obtained wastewater with Ca(OH)_2 _(up to have its pH equal to 6.5), urea (0.45 g/l) and then 1 N NaOH (to adjust its pH to 7.8 ± 0.2). AOMW was placed in 4 l glass jars, where it was vigorously mixed (through a magnetic stirrer) and purged with 0.22 μm filter (Millipore, MO, USA)-sterilized O_2_-free N_2 _at room temperature for 3 h, before being employed in the experiments. AOMW COD and total phenol concentration were about 15 and 0.8 g/l, respectively.

### Bioreactors, their inoculation, working conditions and sampling

The bioreactor system employed in the study was composed by a GAC-packed bed anaerobic biofilm reactor hydraulically connected to an aerobic SB-packed bed biofilm reactor. AOMWs were continuously introduced in the first reactor and allowed to undergo sequential anaerobic biomethanisation and aerobic decontamination (Fig. 1).

Configuration, development procedure and working conditions of the GAC-anaerobic biofilter are reported in Bertin et al. [8], where this innovative bioreactor system has been described in detail. In brief, it was a 2.400 l, hermetically closed and thermostated glass column reactor equipped with a recycle line, an AOMW inlet line at the bottom and an outlet line (for treated wastewater and produced biogas) departing from the top and reaching a closed reservoir hydraulically connected to a 4 l "Mariotte" bottle. After its packing with GAC (its working volume became 1.032 l) it was inoculated with the anaerobic, OMW-digesting microbial consortium developed by Beccari et al. [12,13] and then employed under strictly anaerobic conditions for 9 months of experiments (see Bertin et al. [8] for more details). Biofilm occurring in the reactor at the end of the study was composed by *Proteobacteria*, bacteria of the *Flexibacter-Cytophaga-Bacteroides *group, sulfate-reducing bacteria, low G+C gram-positive bacteria and, in a minor extent, *Methanobacterium formicicum *[8].

The aerobic reactor consisted of a 0.7 l glass column reactor with an external jacket in which water at 30°C was continuously recycled (Fig. 1). The inlet line and the line for supplying 0.22 *μ*m filter-sterilized air were at the bottom of the column, whereas the outlet lines for exhaust air and treated wastewater were placed on a small reservoir located at the top of the reactor. A recycle line continuously carried wastewater from such a reservoir to the bottom of the reactor. In the same reservoir, a probe for dissolved oxygen (97–08 model, ATI-Orion, Boston, MA) and a probe for pH (81–04 model, ATI-Orion, Boston, MA) were also placed. The bioreactor system, sterilised by recycling an aqueous ethanol solution (70 % v/v) containing HCl (1% v/v) for 2 days, was washed with sterile water and then packed with 213 g (dry weight) of SB previously sterilised in autoclave (110°C per 30 min). The developed reactor was made aerobic by supplying sterile air at 60 ml/min and then fed with the effluent of anaerobic digestor. Considering the medium displacement due to the support (0.330 l) and the supplied air (0.020 l), the actual reactor working volume was 0.350 l. The reactor was allowed to operate in batch mode at high recycling flow rate (upflow; at 0.030 l/min) for 2 weeks to permit a preliminary native biomass adhesion on SB surface. Then, it was forced to operate under continuous mode, and this by feeding it with the anaerobic digested wastewater at the same rate at which it was produced by the anaerobic digestor.

The sequential anaerobic-aerobic biofilter system was fed with AOMW at a high and constant organic load (calculated by multiplying COD or total phenol content of the influent by the dilution rate at which each reactor operated) for a 2-month period (Table 1). In particular, the anaerobic GAC-digestor was fed at a dilution rate (D, expressed as the ratio between wastewater influent flow rate and the reactor reaction volume) of 2.077 day^-1 ^with COD and total phenol loading rates of about 31.74 and 1.70 gl^-1^day^-1^, respectively. Given the AOMW decontamination efficiency achieved in the anaerobic digestor, such operative conditions imposed to the aerobic biofilter to operate at a D of 6.171 day^-1 ^and with COD and total phenol loading rates of 50.42 and 2.04 gl^-1^day^-1^, respectively (Table 1). The recycle flow rate of each reactor was set up as a function of D to have a recycle ratio (defined as the ratio of the returned flow rate to the influent flow rate) of 77 for both reactors.

Six ml samples of wastewater were taken daily through sampling ports placed along the inlet line of the integrated reactor system, the inlet and outlet lines of the aerobic reactor (Fig. 1). The collected samples were filtered on 0.22 μm cellulose-nitrate filters (Millipore, MO, USA) and then analysed for COD, the concentration of total phenols and that of volatile fatty acids as detailed in previous papers [7,8]. An aliquot of each sample was also analysed for low molecular weight phenols and aromatic compounds by HPLC [23]. At the end of the 2 month-experiment, the aerobic reactor was opened and triplicate 3 g-samples of SB carrier were collected at 5, 20 and 38 cm of height (from the bottom) of the reactor packed-bed and subjected to gravimetric measurements of immobilized biomass. To this aim, the carriers were gently washed with distilled water, dried at 105°C for 16 h, weighted and then suspended in a 1 M NaOH solution at 90°C for 20 min to induce the releasing of the attached biomass. Again, the biomass free carriers were washed with distilled water, dried at 105°C for 16 h and weighted. The biomass weight was calculated as the weight difference of the dried carrier before and after the NaOH treatment. A second set of SB samples (of about 20 g each) collected from the same regions of the reactor were washed and subjected to DNA extraction as described below.

### DNA extraction

DNA was extracted from the biofilm, influent and effluent samples by using the DNeasy tissue kit (Qiagen, Italy) as described previously [8]. The amount and quality of nucleic acids were checked by electrophoresis on an ethidium bromide-stained 1% agarose gel.

### 16S rRNA amplification and T-RFLP analysis

16S rRNA genes from the extracted DNA samples were amplified with universal eubacterial primers 63F and 1389R [49]. The primer 63F was labeled with 6-FAM (5-[6]-carboxy-fluorescein) on the 5'-end (Applied Biosystems, Italy). Fluorescently labeled PCR products (100 ng) were digested with 10 U of restriction enzyme (Invitrogen, Italy) at 37°C for at least 4 h. T-RFLP profiles were generated using the restriction enzyme *Rsa*I. Additional profiles were generated using the restriction enzyme *Hha*I in order to confirm results obtained with *Rsa*I, and to assist in the assignment of tentative phylogenetic affiliations to T-RFs. The digested samples were run on an ABI Prism 310 Genetic Analyzer (Applied Biosystems) operating in a GeneScan mode with filter set D and the sizes of fragments were compared with internal standards. Replicate T-RF profiles gave reproducible fingerprints.

### DNA sequencing and phylogenetic analysis

Partial clone libraries of 16S rRNA genes were generated from community samples. Unlabeled PCR products, purified as described above, were cloned using the pGEM-T easy vector system (Promega) and *Escherichia coli *JM109 according to the manufacturer's instructions. From each library randomly selected clones were screened for positive inserts and by T-RF analysis using *Rsa*I and *Hha*I restriction enzymes for digestion. Thirtyfour clones from the bacterial libraries were subjected to cycle sequencing using the M13 primers and the BigDye terminator cycle sequencing ready reaction kit (Applied Biosystems). The DNA sequences were bi-directionally resolved on an ABI Prism 310 in a sequencing mode. Nucleotide sequences were checked for potential chimeric sequences using the CHIMERA-CHECK software, and compared with the sequences in the Ribosomal Database Project (RDP) database to identify the closest relatives. The phylogenetic analysis was carried out according to the maximum likelihood method and neighbor-joining topology using the appropriate tools of the RDP program package. Bootstrapping using 1000 replicates was performed to test reliability of the branches of the trees.

## Competing interests

The author(s) declare that they have no competing interests.

## Authors' contributions

LB carried out the experimental work concerning the performances of the integrated anaerobic-aerobic biofilter system, MCC carried out the experimental work concerning the molecular characterization of biofilm developed in the aerobic post-treatment, MR coordinated the latter research activity as well as the manuscript preparation, LM coordinated the biotechnological process research activity and FF coordinated the biotechnological process research activity as well as the manuscript preparation. All authors read and approved the final manuscript.
